# A Caustic Hydrochloric Acid Enema: A Case Report

**DOI:** 10.7759/cureus.35394

**Published:** 2023-02-24

**Authors:** Tom Saliba, Denis Tack

**Affiliations:** 1 Radiology, Université Libre de Bruxelles, Brussels, BEL; 2 Radiology, Centre Hospitalier EpiCURA - site de Ath, Ath, BEL

**Keywords:** muriatic acid, hydrochloric acid, rectitis, caustic rectitis, caustic enema

## Abstract

Rectitis caused by the administration of a caustic enema is uncommonly encountered in routine clinical practice. The reasons given for the application of caustic enemas are diverse, including but not limited to suicide attempts, murder attempts, iatrogenic causes and simple mistakes. When caustic enemas do occur the consequences can be dire, resulting in extensive injury. These injuries often prove fatal in the short term, but if the patient survives the initial injuries, they may subsequently cause severe disability. Treatment can be conservative but commonly involves surgery, with a significant proportion of patients not surviving the intervention or succumbing to complications thereafter. We present the case of a patient with a history of alcoholism, depression and a recent recurrence of oesophageal cancer who self-administered a hydrochloric acid enema in an attempted suicide. The patient subsequently suffered a stenosis of the lower bowel, resulting in diarrhoea. A colostomy was performed in order to alleviate the patient's symptoms and improve their comfort.

## Introduction

Caustic enemas are an uncommon cause of rectitis, with only a few cases reported in the literature [1-3]. When a patient presents with such an ailment, a good anamnesis is essential in revealing the cause as symptoms are non-specific [1-3]. If left untreated, this pathology may easily prove fatal [1-3].

## Case presentation

**Figure 1:**
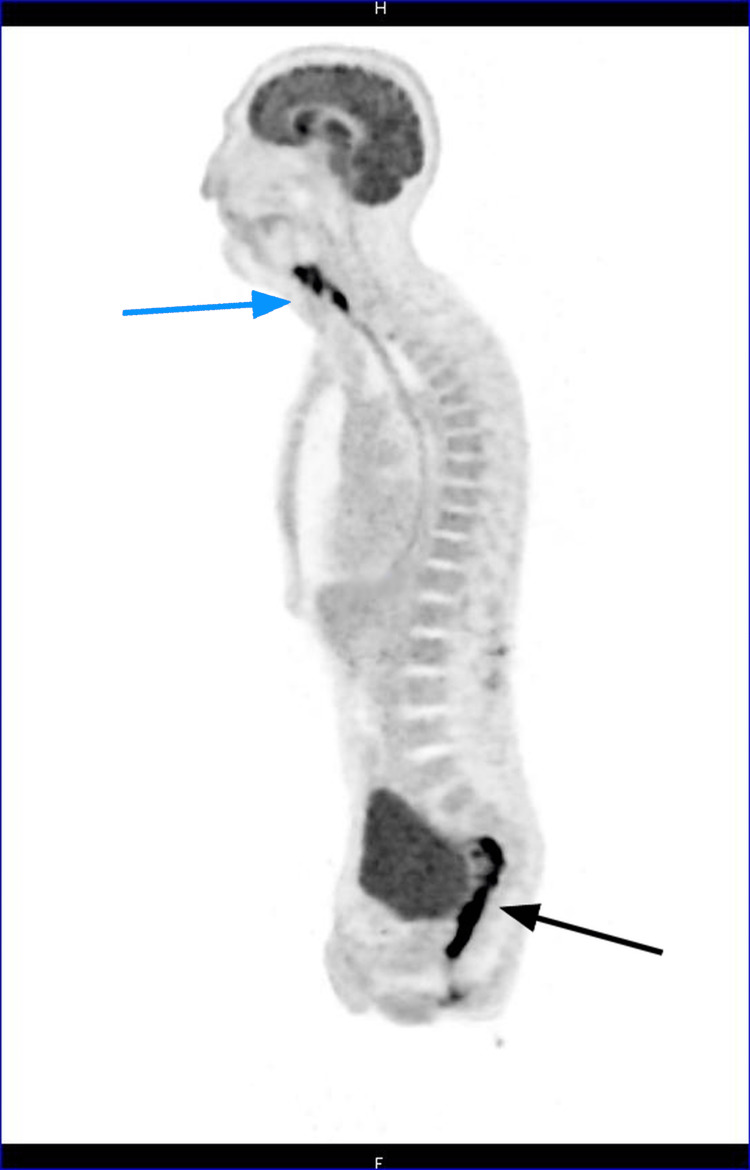
Sagittal PET-CT exam reconstructions showing extensive hyperactivity along the affected bowel segment (black arrow) and high activity zones in the neck (blue arrow) due to cancerous relapse. PET-CT: positron emission tomography-computed tomography

**Figure 2:**
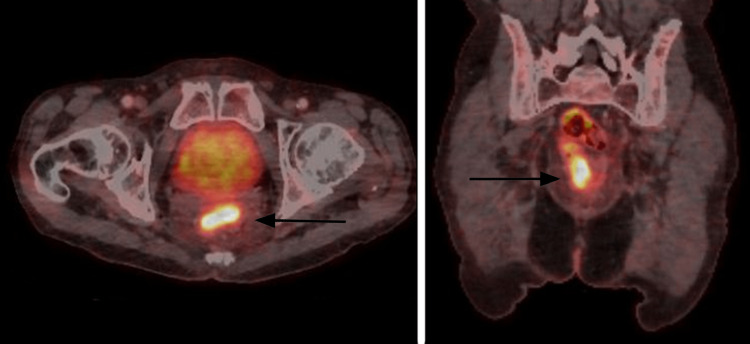
Axial and coronal reconstructions of a PET-CT exam centred on the pelvis showing high activity zones (black arrows) due to the inflammation caused by the caustic enema. PET-CT: positron emission tomography-computed tomography

**Figure 3:**
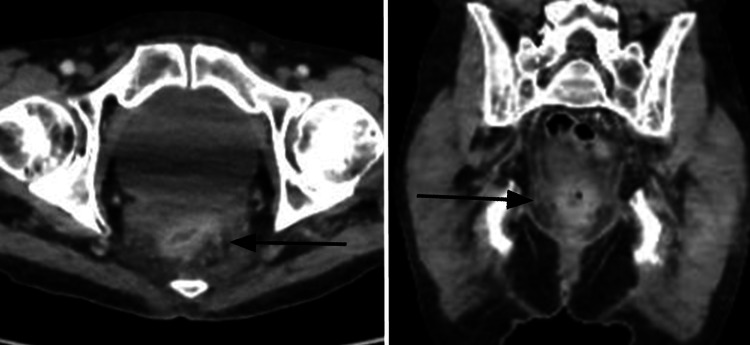
Axial and coronal reconstructions of a CT exam centred on the pelvis showing thickening of the rectal tissue, inflammatory infiltration of the perirectal fat and associated mucosal hyperaemia (black arrows). This is suggestive of a pathology of inflammatory and not oncological origin. CT: computed tomography

A 53-year-old man presented to the emergency department with severe anal pain. The patient was an alcoholic, known to be living in difficult social circumstances, who was suffering from depression with suicidal ideation and had recently suffered a relapse of oropharyngeal cancer. As a result, the patient decided to instil the spirit of salt, otherwise known as hydrochloric acid, into his rectum in an attempt to end his life. This resulted in caustic burns to the anus, rectum and colon, causing the patient a tremendous amount of pain and precipitating his arrival at the emergency department. The gastroenterologist examined the patient and attempted to perform a digital rectal exam but was unable to reach beyond the anorectal junction. Further assessment of the extent of the injury by way of an endoscope was attempted but it was that the lesion was impassable due to it having transformed into fibrotic stenosis. Biopsies of the fibrotic lesion found inflammatory tissue. The positron emission tomography-computed tomography (PET-CT) exam revealed high activity zones around the oropharynx, the seat of cancer, as well as high activity in and around the anus, rectum and distal sigmoid colon (Figures 1-2). The CT exam showed infiltration around the affected zones (Figure 3). In the weeks after the incident the patient underwent psychiatric counselling, after which he began diazepam treatment in an attempt to diminish his alcohol consumption as well as beginning escitalopram treatment for his depression. Due to the stenosis of the lower bowel, resulting in diarrhoea, the decision was made to perform a colostomy in order to alleviate the symptoms and improve the quality of life of the patient. The laryngeal cancer was treated by laryngectomy and glossectomy, which was preceded by a gastrostomy to allow the patient to be fed.

## Discussion

Caustic rectitis is a rare cause of rectal damage, with only a few cases reported in the literature. Caustic rectitis caused by hydrochloric acid administration is rarer still being the chemical used in very few cases [1,2]. When caustic rectitis occurs, the reasons for its administration are varied. In previous reports, caustic enemas resulted from suicide attempts, abortion attempts, murders, self-medication for constipation and mistaken identity of the product [1,2]. In one case series, many of those who attempted suicide had psychological comorbidities [2]. Interestingly, a relatively common cause of caustic rectitis is iatrogenic, due to insufficient washing of endoscopes after cleaning with glutaraldehyde, with residues of the substance causing colitis [4]. Other types of enemas which have resulted in injury found in the literature include sulfuric acid, potash, herbal remedies, hydrogen peroxide, acetic acid, hydrofluoric acid and ammonia [1-3].

Due to the rarity of the entity, the discussion will cover caustic enemas in general. The symptomatology is varied, though abdominal pain, often accompanied by a burning sensation, anorectal burning, rectal bleeding, vomiting, rejection of necrotic tissue and mucus, hypotension and a hyper or hypotonic anal sphincter have all been reported [1-3]. Of note is the fact that there are no reports of peritonitis due to caustic enemas [2,3].

Patients presenting after caustic enema administration should be assessed for intestinal bleeding which, if present, may require hemodynamic stabilisation [3]. One should consider abdominal and chest imaging to search for free air due to the possibility of perforation [3]. Once the absence of peritoneal findings is established, an endoscopic technique should be used to determine the extent of the damage, though some suggest that this may be of little use or even dangerous due to the increased risk of perforation [2,3]. In the case of our patient, endoscopy was attempted but proved impossible due to rectal stenosis. The injuries caused can be extensive and diverse, ranging from damage to the rectum to all areas of the colon, with some reports of damage extending into the ileum [2,3].

Delayed findings include stenosis, necrosis, toxaemia, fistula and peritonitis as well as less severe symptoms such as pain and rectal bleeding [2].

Treatment options are diverse. Conservative treatments include fasting, antibiotics, corticotherapy and parenteral nutrition, though no studies evaluating their efficacy currently exist [3]. If conservative treatment fails surgery may be attempted, often involving the resection of the damaged intestinal tissue, with associated mortality being over 50% in some case series [2,3]. If the patient survives long enough to develop stenoses these may be treated with dilatation [2,3]. If dilatation of the stenoses fails then resection of the aforementioned segments may be attempted [2,3].

Of note is the fact that many reports come from Africa, with Diarra et al. suggesting that this is due to the widespread use of intrarectal injections [2]. Diarra et al. explain that this is due to intrarectal medications being commonplace in sub-Saharan African traditional medicine, making it part of daily practice in those communities [2].

## Conclusions

Although mostly encountered in countries in Africa, where enemas are more commonly used than in western countries, patients having undergone a caustic enema may in rare cases present to any emergency department. The presentation is often non-specific, generally with symptoms of rectal and abdominal pain, with diagnosis relying on anamnesis. The treatment options vary widely, ranging from conservative treatment such as antibiotics and corticosteroids to surgical interventions. Even if treated, mortality and morbidity are high due to gastrointestinal complications. We presented an atypical case of rectitis due to a hydrochloric acid enema, resulting in anorectal stenosis, requiring surgical treatment to help alleviate the symptoms. By virtue of this case report, we hope to bring attention to this rare cause of rectitis in order to encourage in-depth anamnesis and history-taking.
